# A Ni(II) Coordination Polymer as a Multifunctional Luminescent Sensor for Detection of UO_2_^2+^, Cr_2_O_7_^2−^, CrO_4_^2−^ and Nitrofurantoin

**DOI:** 10.3390/molecules28124673

**Published:** 2023-06-09

**Authors:** Yun-Shan Xue, Xin-Yue Zhang, Zheng-Chen Tian, Jing-Rui Cao, Wen-Jing Wang, Ru-Xiu Tang, Jie Guo, Zheng-Hao Fei, Jun Wang

**Affiliations:** School of Chemistry & Environmental Engineering, Yancheng Teachers University, Yancheng 224007, China

**Keywords:** luminescent sensor, UO_2_^2+^ sensing, Cr_2_O_7_^2−^/CrO_4_^2−^ sensing, nitrofurantoin sensing, coordination polymer

## Abstract

A new Ni coordination polymer [Ni(MIP)(BMIOPE)]_n_ (**1**) was constructed (BMIOPE = 4,4′-bis(2-methylimidazol-1-yl)diphenyl ether, and H_2_MIP = 5-methylisophthalic acid), possessing two-dimensional (2D) twofold parallel interwoven net structure with a 4^4^∙6^2^ point symbol. Complex **1** has been successfully obtained based on mixed-ligand strategy. The fluorescence titration experiments revealed that complex **1** could act as multifunctional luminescent sensor to simultaneously detect UO_2_^2+^, Cr_2_O_7_^2−^ and CrO_4_^2−^, and NFT (nitrofurantoin). The limit of detection (LOD) values for complex **1** are 2.86 × 10^−5^, 4.09 × 10^−5^, 3.79 × 10^−5^ and 9.32 × 10^−5^ M for UO_2_^2+^, Cr_2_O_7_^2−^, CrO_4_^2−^ and NFT. The K_sv_ values are 6.18 × 10^3^, 1.44 × 10^4^, 1.27 × 10^4^ and 1.51 × 10^4^ M^−1^ for NFT, CrO_4_^2−^, Cr_2_O_7_^2−^ and UO_2_^2+^. Finally, the mechanism of its luminescence sensing is studied in detail. These results manifest that complex **1** is a multifunctional sensor for sensitive fluorescent UO_2_^2+^, Cr_2_O_7_^2−^, CrO_4_^2−^ and NFT detection.

## 1. Introduction

Uranium, a naturally occurring heavy metal element, can be used as nuclear fuel and plays a crucial part in the nuclear power industry. However, uranium contamination is easily caused by nuclear accidents and various nuclear industries such as uranium mining and processing and nuclear power plants. Owing to its high radioactivity and chemical toxicity, it has also aroused worldwide concern about potential risks to human health. The stable form of uranium in aqueous solution is uranyl ion (UO_2_^2+^). Excessive accumulation of UO_2_^2+^ in the human body may cause irreversible damage to the kidney, liver and other important organs as well as the immune system, leading to an increased risk of cancer [1]. According to the United States Environmental Protection Agency (EPA), the permissible value of UO_2_^2+^ in drinking water should not exceed 35 μg L^−1^ [2]. In recent years, the detection of uranium has attracted more attention. As is known to all, chromium (VI) is widely used in the leather tanning, chromium plating, printing and dyeing industries. It also produces a large amount of chromium-containing wastewater. Heavy metal ions are not able to be degraded directly and easily accumulate in ecosystems. For example, CrO_4_^2−^ and Cr_2_O_7_^2−^, with high toxic in water, will cause serious harm to human health after entering the human body because of their carcinogenicity [3]. On the other hand, Nitrofurantoin (NFT) is a broad-spectrum nitrofuran antibiotic widely used in aquaculture and animal husbandry to treat protozoan and bacterial infections. However, abused or blindly added NFT may result in increased bacterial resistance and environmental pollution; there is no doubt that this is a large threat to ecological environments and human health [4]. Thus, the trace detection of UO_2_^2+^, CrO_4_^2−^, Cr_2_O_7_^2−^ and NFT from natural water or wastewater has been a matter for concern.

Conventional detection methods have been used to detect these pollutants, such as atomic absorption spectrometry (AAS), inductively coupled plasma mass spectrometry (ICP-MS), X-ray fluorescence spectrometry (XRF) and so on. However, these technologies are readily available and costly and cannot be detected in real time and on the spot. Hence, exploring and developing simple, fast and accurate methods is essential to detect toxic organic solvents, metal cations and anions [5]. 

Coordination polymers (CPs), as a novel class of crystalline porous materials, have been demonstrated to have high potentials in many fields such as fluorescent sensing, delivering biomedicine, gas storage, molecular recognition, catalysis and so on, on account of specific surface area, functional pore structures and adjustable atom-precise guest-host structures [6,7,8,9,10]. Recently, luminescent CPs as a kind of chemical sensors have been widespread used to monitor various environmental pollutants including toxicity metal ions, toxic inorganic anions, nitro explosives and antibiotics on account of their remarkable advantages such as on-site detection, high selectivity, sensitivity and low detection limit [11,12,13,14]. Many researchers reported the use of LCPs to detect Fe^3+^, Pb^2+^, Al^3+^, Hg^2+^, Cr_2_O_7_^2−^, CrO_4_^2−^ and other small organic molecules with high sensitivity. However, there are only few reports of LCPs to detect UO_2_^2+^ by fluorescence quenching. Ye et al. reported an anionic Tb-MOF as dual-channel luminescence probe for UO_2_^2+^ ion [15]. Hou et al. reported a carboxyl-functionalized Zn-MOF as bi-functional chemical probe for sensing trace amounts of Pb^2+^ and UO_2_^2+^ ions in aqueous solution [16]. Chen et al. reported a Co-MOF with exposed pyrimidyl Lewis base sites as fluorescent sensor for UO_2_^2+^ and Al^3+^ [17]. These CPs based on Lanthanide metals and transition metals exhibit detection performance for UO_2_^2+^. However, it is still a great challenge to improve the fluorescence response and sensitivity of CP-based sensors, which also need to be further researched and developed. At present, few CPs can simultaneously detect UO_2_^2+^, Cr_2_O_7_^2−^, and CrO_4_^2−^ and NFT [18]. 

Inspired by the aforementioned viewpoints, we report a new Ni(II) CP [Ni(MIP)(BMIOPE)]n (**1**), while H_2_MIP = 5-methylisophthalic acid, and BMIOPE = 4,4′-bis(2-methylimidazol-1-yl)diphenyl ether (Scheme S1). Complex **1** exhibits 2D twofold parallel interwoven net. A series of the fluorescent detection experiments have found that complex **1** could be a high-efficiency multi-responsive sensor by fluorescence quenching to detect low concentrations of UO_2_^2+^ cation, Cr_2_O_7_^2−^/CrO_4_^2−^ anions and NFT antibiotic with noteworthy selectivity and high sensitivity. Furthermore, the possible sensing mechanisms were studied in detail. 

## 2. Results and Discussion

### 2.1. Structure Description of [Ni(MIP)(BMIOPE)]_n_ (1)

**Figure 1:**
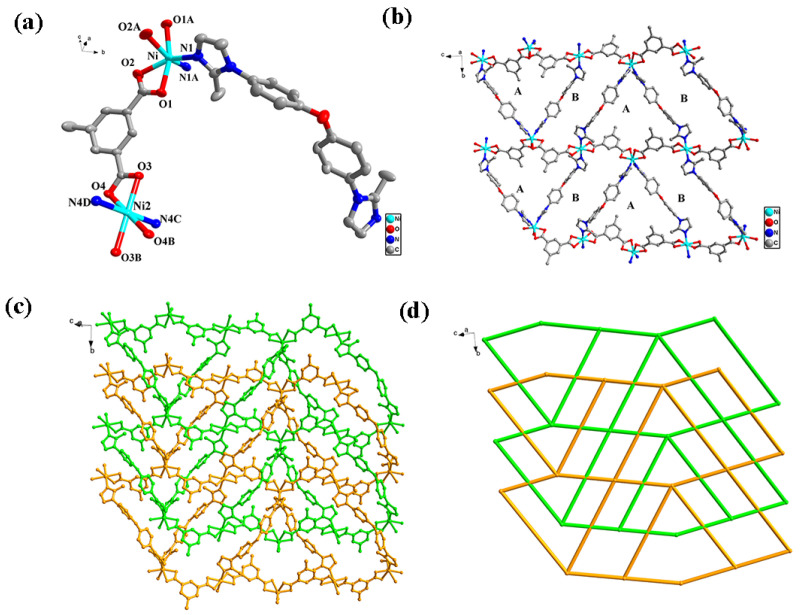
(**a**) Coordination environment of the central Ni atom; (**b**) a single 2D network of complex **1** containing cavities A and B; (**c**) the representation of the twofold interpenetrating layers of complex **1**; (**d**) a schematic of the twofold interpenetrated topology.

Structure analysis shows that complex **1** crystallizes in monoclinic crystal system with space group *C*2/*c* by employing X-ray single crystal diffraction. The asymmetric unit consists of two independent Ni^2+^ ions (Ni1, Ni2), one MIP^2−^ organic linker and one BMIOPE organic linker. From the view of Figure 1a, Ni1 ions display six-coordinated octahedral configuration, consisting of four O atoms (O1, O1A, O2, O2A) from two MIP^2−^ linkers and two N atoms (N1 and N1A) from two BMIOPE linkers (Ni1-O1 = 2.0775(15) Å, Ni-O2 = 2.200(2) Å, Ni1-O1A = 2.0775(15) Å, Ni-O2A = 2.200(2) Å, Ni1-N1 = 2.048(2) Å, Ni1-N1B = 2.048(2) Å, symmetry code: A 1 − x, +y, 1.5 − z). Ni2 ions are six-coordinated by four O atoms (O3, O4, O3B, O4B) from two MIP^2−^ organic linkers and two N atoms (N4C and N4D) from two BMIOPE organic linkers, forming an octahedral configuration (Ni2-O3 = 2.0759(14) Å, Ni2-O3B = 2.0759(14) Å, Ni2-O4 = 2.1521(16) Å, Ni2-O4B = 2.1521(16) Å, Ni2-N4C = 2.1208(19) Å, Ni2-N4D = 2.1208(19) Å, symmetry code: B 0.5 − x, −0.5 − y, 1 − z, C 0.5 − x, 1.5 − y, 1 − z, D +x, −2 + y, +z). In complex **1**, neighbor Ni1 and Ni2 ions are connected to MIP^2−^ ligands to from *zigzag* one-dimensional (1D) chains [Ni(MIP)]_n_ with Ni1∙∙∙Ni2 range of 9.065 Å (Figure S1a), while BMIOPE ligands connect adjacent Ni1 and Ni2 ions to generate *zigzag* 1D chains [Ni(BMIOPE)]_n_ with a Ni1∙∙∙ Ni2 separation of 16.522 Å (Figure S1b). These two zigzag chains are interconnected by Ni1 and Ni2 ions to generate a 2D layer arranged parallel along the [1 0 −1] direction. Two kinds of large cavities have the maximum dimensions of 15.622 Å × 17.795 Å (type A) and 15.622 Å × 21.568 Å (type B) in each net calculated on the diagonal Ni∙∙∙Ni separation (Figure 1b). Such large cavities allow the other net to penetrate and form twofold parallel interwoven net (Figure 1c). Topologically, Ni1 and Ni2 ions act as four connecting nodes, and complex **1** is a 4^4^∙6^2^ 4-connected uni-nodal network (Figure 1d). The calculated free volume in complex **1** is 2.5% (per unit crystal volume of 5336.1 Å^3^) calculated using PLATON. The framework of complex **1** is further stabilized by weak but extensive inter- and intra-molecular C–H∙∙∙O hydrogen bonds connecting the 2D layers into a 3D hydrogen-bond structure.

### 2.2. PXRD and Thermal Analyses

The powder XRD pattern for complex **1** is shown in Supplemental Figure S2. In comparison with the simulated one from crystal structure analysis, the main peak patterns of complex **1** match very well, indicating single phase purity of the bulk sample. In addition, thermal stability was investigated by using thermal analyzer in temperature range of 25–800 °C under the N_2_ atmosphere. The curve displayed that the skeleton of complex **1** began to collapse after ca. 440 °C and displayed relatively good thermal stability (Figure S3).

### 2.3. Photoluminescence Properties

Solid-state luminescence properties of complex **1** were tested at room temperature. The center of the emission bands appears at 357 nm (λ_ex_ = 315 nm) and 363 nm (λ_ex_ = 303 nm) for H_2_MIP and BMIOPE, respectively, which might be ascribed to *n*/*π*→π* electron transitions [19,20]. Complex **1** displays the emission maxima centered at 346 nm upon excitation at 245 nm, as exhibited in Supplemental Figure S4. Since the fluorescence intensity of carboxylate ligands is very weak compared with that of nitrogen-containing ligands, the fluorescence of complex **1** may be ascribed to the intra-ligand transitions in BMIOPE ligand [21,22]. To further verify the fluorescence properties of complex **1**, the fluorescence quantum yield of complex **1** and the ligand BMIOPE were measured at room temperature. The fluorescence quantum yield of the ligand BMIOPE was 3.58%. The fluorescence quantum yield of complex **1** was measured under the same conditions, and the results showed that the fluorescence quantum yield of complex **1** was 8.12%. The fluorescence quantum yield is improved, mainly because the ligand is better fixed to form a more stable rigid structure after the metal ion connected with the ligand, which hinders its torsion and leads to the improvement of fluorescence yield.

### 2.4. Selective Detection of UO_2_^2+^ Cations

**Figure 2:**
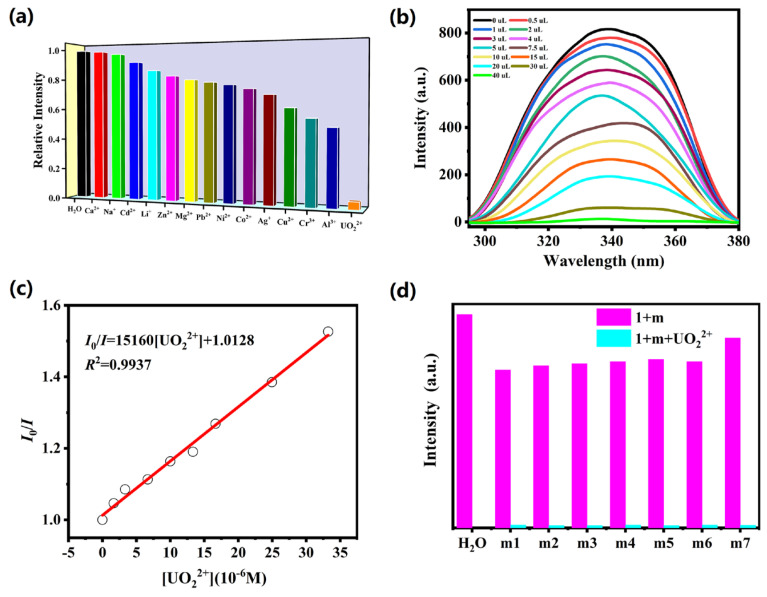
(**a**) The relative intensity of **1** in aqueous solutions of diverse ions (10 mM); (**b**) the fluorescence intensity of **1** upon incremental addition of UO_2_^2+^ ion; (**c**) Stern−Volmer plots of UO_2_^2+^ in **1**; (**d**) the fluorescence intensities of **1** with different mixed ions solution added UO_2_^2+^ ion (m1: Ca^2+^/Al^3+^; m2: Na^+^/Cr^3+^; m3: Cd^2+^/Cu^2+^; m4: Li^+^/Ag^+^; m5: Zn^2+^/Co^2+^; m6: Mg^2+^/Ni^2+^; m7: Pb^2+^).

Considering the excellent luminescence performance, sensing properties of complex **1** towards metal ions were explored. The powder sample of **1** (5 mg) was added into different aqueous solution containing M(NO3)n (M = Na^+^, Ag^+^, Li^+^, Cd^2+^, Zn^2+^, Pb^2+^, Co^2+^, Ca^2+^, Cu^2+^, Ni^2+^, Mg^2+^, Al^3+^, Cr^3+^ and UO_2_^2+^, 10 mM), respectively. The emission spectra of complex **1** is the metal ion-dependent, and UO_2_^2+^ ion shows the significant fluorescence quenching effects on the emission intensities of complex **1** (Figure 2a). So, complex **1** could be used to selectively detect UO_2_^2+^ in aqueous solutions. Thus, to explore the sensing sensitivity to detect UO_2_^2+^, titration experiments were implemented to explore the intensities of complex **1** with increasing amounts of UO_2_^2+^ ionic solutions. With increasing solution volume of UO_2_^2+^, the intensities of **1** continuously decrease (Figure 2b). Stern–Volmer (SV) formula *I*_0_/*I* = 1 + *K*_SV_[Q] was used to analyze the quenching efficiency (*I*_0_ and *I* mean the emission intensities prior and after the addition of analytes; [Q] means the concentration of the analytes; *K*_SV_ refers to the quenching coefficient). Figure 2c presents the Stern–Volmer plots: the *I*_0_/*I* is linearly proportional to UO_2_^2+^ concentration (*R*^2^ = 0.9937) at low concentrations, and the slope K_SV_ for UO_2_^2+^ is quantified to be 1.51 × 10^4^ M^−1^, confirming high sensitivity of complex **1** toward UO_2_^2+^. The limit of detection (LOD) could be estimated by the formula 3*σ*/*K*_SV_ (*σ* means the standard deviation). From Table S2, the LOD value for UO_2_^2+^ ion is calculated to be 2.86 × 10^−5^ M, which is higher than those reported by CP-based sensors. In order to measure the influence of other metal ions on the detection of UO_2_^2+^, interference experiments were also performed, as depicted in Figure 2d. It is found that the addition of other competing ions only reduced the emission intensity of complex **1** to some extent, but the intensity was almost quenched after the addition of UO_2_^2+^ ions. The results indicate that the presence of other competing ions does not disturb the detection of complex **1** toward UO_2_^2+^. In comparison with other CPs-based sensors for detecting UO_2_^2+^ ions, complex **1** has good detection and analysis performance [16,17,23].

### 2.5. Selective Detection of Cr_2_O_7_^2−^/CrO_4_^2−^ Anions

**Figure 3:**
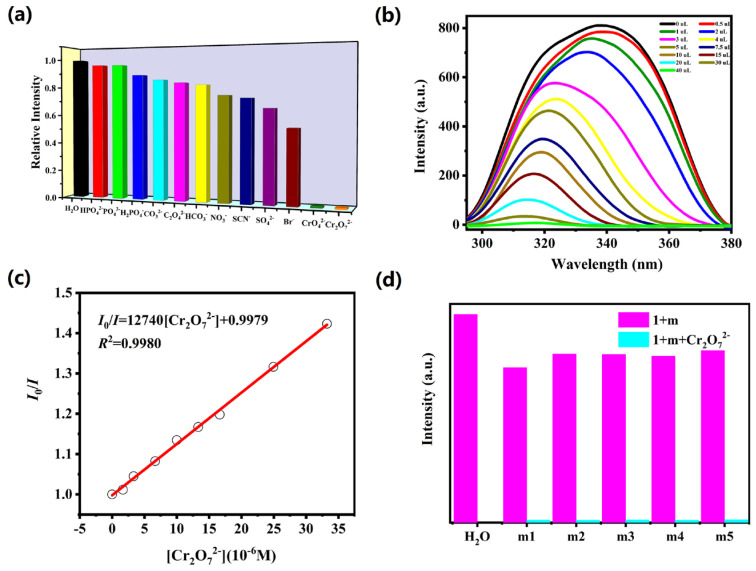
(**a**) The relative intensity of complex **1** in 10 mM different anion solutions; (**b**) the fluorescence intensity of **1** upon incremental addition of Cr_2_O_7_^2−^ ion; (**c**) Stern−Volmer plots of Cr_2_O_7_^2−^ in **1**; (**d**) the emission intensities of **1** with different mixed ions solution added Cr_2_O_7_^2−^ ion (m1: HPO_4_^2−^/Br^−^; m2: PO_4_^3−^/SO_4_^2−^; m3: H_2_PO_4_^−^/SCN^−^; m4: CO_3_^2−^/NO_3_^−^; m5: C_2_O_4_^2−^/HCO_3_^−^).

**Figure 4:**
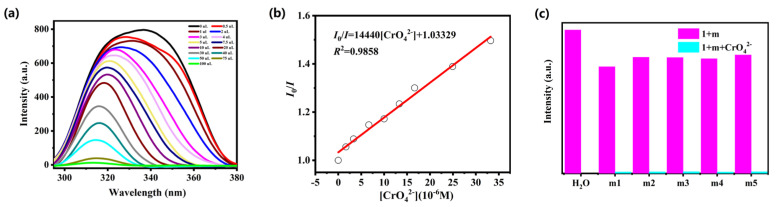
(**a**) The emission intensity of complex **1** upon incremental addition of CrO_4_^2−^ ion; (**b**) Stern−Volmer plots of CrO_4_^2−^ in **1**; (**c**) luminescence intensities of complex **1** with various mixed ions solution added CrO_4_^2−^ ion (m1: HPO_4_^2−^/Br^−^; m2: PO_4_^3−^/SO_4_^2−^; m3: H_2_PO_4_^−^/SCN^−^; m4: CO_3_^2−^/NO_3_^−^; m5: C_2_O_4_^2−^/HCO_3_^−^).

The detection ability of complex **1** towards different anions was explored under the same experimental procedure as UO_2_^2+^. The powder sample of complex **1** (5 mg) was evenly dispersed in 10 mM aqueous solution of Na_n_X (CO_3_^2−^, NO_3_^−^, Br^−^, HCO_3_^−^, SCN^−^, C_2_O_4_^2−^, HPO_4_^2−^, PO_4_^3−^, SO_4_^2−^, H_2_PO_4_^−^, CrO_4_^2−^ and Cr_2_O_7_^2−^) to explore its sensing abilities toward various anions. As demonstrated in Figure 3a, the emission intensities of complex **1** are obviously quenched by Cr_2_O_7_^2−^ and CrO_4_^2−^ ions while other anions show no obvious quenching effect, indicating that Cr_2_O_7_^2−^ and CrO_4_^2−^ ions could be detected by complex **1**. To further explore the fluorescent sensitivities of complex **1** toward Cr_2_O_7_^2−^ and CrO_4_^2−^ anions, titration experiments were performed in aqueous solution. The luminescent intensities of complex **1** gradually decreased with the increasing concentration of Cr_2_O_7_^2−^ and CrO_4_^2−^ anions (Figure 3b and Figure 4a). The *K*sv values are 1.27 × 10^4^ M^−1^ (*R*^2^ = 0.9980) for Cr_2_O_7_^2−^ and 1.44 × 10^4^ M^−1^ (*R*^2^ = 0.9858) for CrO_4_^2−^, as depicted in Figure 3c and Figure 4b. The LOD values for Cr_2_O_7_^2−^ and CrO_4_^2−^ anions are 4.09 × 10^−5^ M and 3.79 × 10^−5^ M (Table S2). Furthermore, we also carried out the interference experiments to evaluate the selectivity of complex **1** against Cr^VI^ (Cr_2_O_7_^2−^/CrO_4_^2−^). As shown in Figure 3d and Figure 4c, the capability of complex **1** to detect Cr_2_O_7_^2−^ and CrO_4_^2−^ anions is not readily interfered by other anions. The sensing capabilities are commensurate with previously reported CP-based luminescent sensors for detection of hexavalent chromate [24,25,26,27].

### 2.6. Selective Detection of NFT

**Figure 5:**
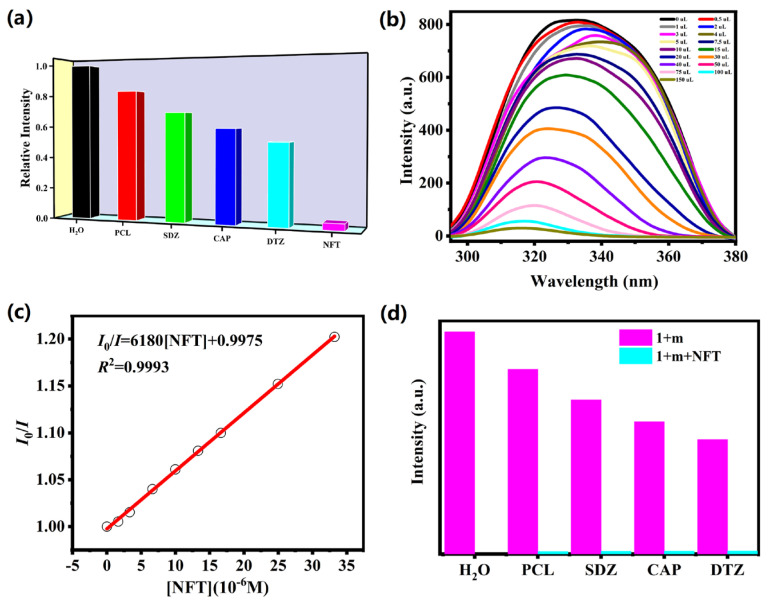
(**a**) The relative emission intensity of complex **1** in 10 mM different antibiotics; (**b**) the intensity of **1** upon incremental addition of NFT; (**c**) Stern−Volmer plots of NFT in **1**; (**d**) the emission intensities of **1** with different antibiotics solution added NFT.

The excellent fluorescence response of complex **1** towards UO_2_^2+^, Cr_2_O_7_^2−^ and CrO_4_^2−^ ions drove us to explore its sensing abilities towards various antibiotics such as PCL (penicillin), NFT (nitrofurantoin), CAP (chloramphenicol), SDZ (sulfadiazine) and DTZ (dimetridazole). From Figure 5a, the intensities of complex **1** were reduced to almost zero only with the addition of NFT, and the results show that complex **1** could be used as possible fluorescence probe to detect NFT with high selectivity. The final quenching efficiency order is as follows: PCL ˂ SDZ ˂ CAP ˂ DTZ ˂ NFT. A series of titration experiments were executed to explore the sensitivity for NFT, which revealed that the emission intensity decreased gradually with adding nitrofurantoin (Figure 5b). As shown in Figure 5c, the curve between the concentrations of NFT and *I*_0_/*I* can be well-fitted by employing the S-V formula (*R*^2^ = 0.9975). The *K*_SV_ and LOD values of complex **1** towards NFT are 6.18 × 10^3^ M^−1^ and 9.32 × 10^−5^ M, respectively, as exhibited in Figure 5c and Supplemental Table S3. Otherwise, the interference experiments manifested that the presence of other antibiotics does not make any significant change in the sensing of NFT, confirming that complex **1** has good anti-interference ability (Figure 5d). The sensing performances of complex **1** are superior to most reported CPs-based luminescent sensing materials for detection of nitrofurantoin [28,29,30,31].

### 2.7. Detection Mechanism

**Figure 6:**
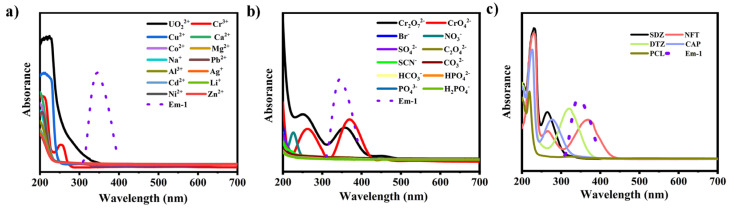
UV-vis spectra of different metal cations (**a**), anions (**b**), antibiotics (**c**) and the emission spectra of **1** (red) in H_2_O solution.

To better understand the strong quenching effect of UO_2_^2+^, Cr_2_O_7_^2−^/CrO_4_^2−^ and NFT on the fluorescence of complex **1**, the quenching mechanism was explored. The fluorescence quenching UO_2_^2+^, Cr_2_O_7_^2−^/CrO_4_^2−^ and NFT caused by the structural collapse was first excluded because the patterns of complex **1** after sensing tests are well matched with the original samples (Figure S2) [32,33]. Additionally, the most significantly overlaps are between the emission spectra of complex **1** and the UV-vis absorption band of the analytes (UO_2_^2+^, Cr_2_O_7_^2−^/CrO_4_^2−^ and NFT), which indicated that there was energy competitive absorption between complex **1** and the analytes, finally causing fluorescence quenching [34,35,36] (Figure 6). According to the density functional theory (DFT), by employing Gaussian 09 with B3LYP/6-31G* method, the HOMO and LUMO energy of the antibiotics was calculated. It is well known that the lower LUMO energy level of antibiotics means the much easier electron transfer from the fluorescent material to antibiotics, leading to the higher fluorescence quenching effects. The order of LUMO energy is PCL ˃ SDZ ˃ CAP ˃ DTZ ˃ NFT, which is in accordance with the quenching efficiency (Supplemental Figure S5 and Table S3). The results indicate that the photo-induced electron transfer (PET) process is pivotal in detecting antibiotics [37,38,39,40]. Thus, the fluorescence quenching of complex **1** toward UO_2_^2+^, Cr_2_O_7_^2−^/CrO_4_^2−^ and NFT is caused by the competitive absorption and PET mechanism.

Additionally, fluorescence lifetime measurements were performed to further investigate the kinetic property of complex **1** toward UO_2_^2+^, Cr_2_O_7_^2−^, CrO_4_^2−^ and NFT. The fluorescence lifetime studies exhibit similar triexponential function-fitted decay curves with varying average lifetimes for the different samples (Figures S6–S10). The fluorescence lifetime of the suspension of complex **1** is 120 ns. With the addition of UO_2_^2+^, Cr_2_O_7_^2−^, CrO_4_^2−^ and NFT, the average lifetimes of complex **1** are 109 ns, 111 ns, 115 ns and 110 ns, respectively. The fluorescence lifetimes of complex **1** show very little change before and after detection of different quenchers, suggesting that the luminescence quenching process should be a static quenching mechanism [41].

## 3. Materials and Methods

### 3.1. Materials and Physical Measurement

All reagents are analytically pure grade in the experiment and are not further purified for use. Thermogravimetric analysis was measured on a NETZSCH STA 449 F5 Jupiter TGA analyzer (Selb, Germany) using an empty Al_2_O_3_ crucible as the standard. Measuring temperature ranges from 25 to 800 °C with a heating rate of 10 °C min^−1^ under N_2_ atmosphere. Powder X-ray diffraction patterns of the title complex were obtained using a Shimadzu XRD-6000 X-ray diffractometer (Kyoto, Japan) with Cu-Kα (λ = 1.5418 Å) radiation at room temperature and 2*θ* ranging from 5° to 50°. Perkin Elmer (Waltham, MA, USA) 240C elemental analyzer was used to obtain elemental analyses of complex **1**. Infrared spectra were obtained on a Bruker VERTEX 80 spectrometer (Billerica, MA, USA) in the 4000–400 cm^−1^ region. Fluorescence data were collected on the Perkin Elmer LS55 Fluorescence Spectrophotometer.

### 3.2. Preparation of [Ni(MIP)(BMIOPE)]_n_ (1)

Ni(NO_3_)_2_·6H_2_O (14.5 mg, 0.05 mmol), 5-methylisophthalic acid (H_2_MIP) (9.1 mg, 0.05 mmol) and 4,4′-bis(2-methylimidazol-1-yl)diphenyl ether (BMIOPE) (16.5 mg, 0.05 mmol) were dissolved in 2.5 mL mixed solvent of water and N,N-dimenthylformamide (V_H2O_:V_DMF_ = 1:1.5). The solution was charged into a Teflon-lined stainless steel vessel, heated from room temperature to 100 °C in 2 h for 3 days under autogenous pressure. The product was then cooled to 40 °C in 2 days and finally cooled to room temperature (yield 56.9%, calculated on Ni(NO_3_)_2_·6H_2_O). Elemental analysis (%) for C_29_H_24_N_4_NiO_5_: C, 61.41; H, 4.26; N, 9.88. Found: C, 61.54; H, 4.27; N, 9.91. IR (KBr, cm^−**1**^): 3130 w, 1606 m, 1548 s, 1508 s, 1440 m, 1382 m, 1302 w, 1238 s, 1173 w, 1107 w, 1005 w, 841 m, 811 w, 774 w, 738 m, 548 w, 443w.

### 3.3. X-ray Crystallography

Crystal X-ray diffraction data for the titled complex was performed on Bruker APEX D8 QUEST diffractometer with a Photon 100 CMOS detector (Mo-Kα radiation, λ = 0.71073 Å). The absorption correction and data processing were conducted through the SADABS and SAINT programs [42]. The structure of the titled complex was solved by direct methods and further refined on *F*^2^ with full-matrix least-squares procedure using the SHELXTL-2014 software package [43]. The non-hydrogen atoms were anisotropically refined, while all hydrogen atoms on carbon atoms were yielded geometrically and refined in the riding mode. Details of the crystallographic data for complex **1** are listed in Table 1. Selected bond lengths and angles for complex **1** are provided in Table S1.

### 3.4. Luminescent Sensing Experiments

The ground powder samples (5 mg) of complex **1** were immersed in 3 mL of water and then ultrasonicated for 1 h to obtain a suspension for luminescence detection. The aqueous solutions of Na_n_X (HCO_3_^−^, CO_3_^2−^, NO_3_^−^, Br^−^, C_2_O_4_^2−^, SCN^−^, HPO_4_^2−^, PO_4_^3−^, SO_4_^2−^, H_2_PO_4_^−^, CrO_4_^2−^ and Cr_2_O_7_^2−^), M(NO_3_)_n_ (M = Li^+^, Na^+^, Ag^+^, Ca^2+^, Cd^2+^, Co^2+^, Zn^2+^, Pb^2+^, Ni^2+^, Mg^2+^, Cr^3+^, Al^3+^, Cu^2+^ and UO_2_^2+^) or different antibiotics (PCL (penicillin), DTZ (dimetridazole), SDZ (sulfadiazine), NFT (nitrofurantoin) and CAP (chloramphenicol)) were employed at a concentration of 10 mM for the qualitative and anti-interference experiments. Titration tests of different concentrations were performed by gradually addition of analyte (UO_2_^2+^, Cr_2_O_7_^2−^, CrO_4_^2−^, NFT) in the aqueous solutions, and then emission spectra were collected. 

## 4. Conclusions

In this work, a novel Ni-CP [Ni(MIP)(BMIOPE)]_n_ (**1**) was synthesized using H_2_MIP and BMIOPE ligands. Complex **1** is a 2D twofold parallel interwoven net structure with a 4^4^∙6^2^ point symbol. Complex **1** could be used as multifunctional luminescent probe toward UO_2_^2+^, Cr_2_O_7_^2−^/CrO_4_^2−^ and NFT with high selectivity and sensitivity in aqueous media. The detection limits of complex **1** were found to be 2.86 × 10^−5^, 4.09 × 10^−5^, 3.79 × 10^−5^ and 9.32 × 10^−5^ M for UO_2_^2+^, Cr_2_O_7_^2−^, CrO_4_^2−^ and NFT, respectively. The quenching mechanism proved that the quenching involved in sensing UO_2_^2+^ cations and Cr_2_O_7_^2−^/CrO_4_^2−^ anions was caused by the energy competitive absorption, while the quenching processes towards NFT might be ascribed to the synergistic effect of the competitive absorption and PET mechanism. The fluorescence lifetime characterization shows that the quenching effect of complex **1** towards UO_2_^2+^, Cr_2_O_7_^2−^/CrO_4_^2−^ and NFT was attributed to static quenching mechanism. 

## Figures and Tables

**Table 1:** Crystal data and structure refinement parameters of complex **1**.

Complex	1
Formula	C_29_H_24_N_4_NiO_5_
Formula weight	567.23
Crystal system	monoclinic
Space group	*C*2/*c*
*a* (Å)	20.4678(11)
*b* (Å)	7.8281(4)
*c* (Å)	34.2314(18)
*α* (^o^)	90
*β* (^o^)	103.369(2)
*γ* (^o^)	90
Volume (Å^3^)	5336.1(5)
*Z*	8
*T* (K)	273(2)
*D*_calcd_ (mg·m^−3^)	1.412
*μ* (mm^−1^)	0.774
*R*_int_	0.0341
*F*(000)	2352.0
*θ* range (°)	2.769 ≤ *θ* ≤ 25.998
Reflns. collected	49,207
Data/restraints/parameters	5254/1/357
Goodness of fit on *F*^2^	1.038
Final *R* indices [*I > 2σ (I)*]	*R*_1_ = 0.0394, w*R*_2_ = 0.0891
*R* indices (all data)	*R*_1_ = 0.0474, w*R*_2_ = 0.0919
Largest diff. peak and hole (e Å^−3^)	0.38 and −0.62

*R*_1_ = Σ| |*F*_o_| − |*F*_c_| |/Σ|F_o_|. *ωR*_2_ = Σ[*w*(*F*_o_^2^* − F*_c_^2^)^2^]/Σ[*w(F*_o_^2^)^2^]^1/2.^

## Data Availability

Crystallographic data for complex **1** have been deposited at the Cambridge Crystallographic Data Centre with CCDC reference number 2247940.

## Supplementary Materials

The following supporting information can be downloaded at: https://www.mdpi.com/article/10.3390/molecules28124673/s1, Table S1. Selected bond lengths (Å) and angles (°) for complex **1**. Table S2. Standard deviation and detection limit calculation of complex **1** for UO_2_^2+^, Cr_2_O_7_^2−^, CrO_4_^2−^ and NFT. Table S3. HOMO and LUMO energy levels of selected antibiotics calculated by density functional theory (DFT) at B_3_LYP/6-31G** level; Scheme S1. Schematic drawing of the ligands BMIOPE and H_2_MIP; Figure S1. (a) View of 1D [Ni(MIP)]_n_ chain in complex **1**; (b) View of 1D [Ni(BMIOPE)]_n_ chain in complex **1**; Figure S2. The PXRD patterns for complex **1** under different conditions; Figure S3. The TG profile of complex **1**; Figure S4. Solid-state excitation and emission spectra of complex **1**; Figure S5. The HOMO and LUMO energy levels for different antibiotics. Figure S6. Fluorescence decay curve for complex **1**. Figure S7. Fluorescence decay curve for complex **1** in the presence of UO_2_^2+^. Figure S8. Fluorescence decay curve for complex **1** in the presence of Cr_2_O_7_^2−^. Figure S9. Fluorescence decay curve for complex **1** in the presence of CrO_4_^2−^. Figure S10. Fluorescence decay curve for complex **1** in the presence of NFT.
