# Supplementary material for: A Ni(II) Coordination Polymer as a Multifunctional Luminescent Sensor for Detection of UO22+, Cr2O72−, CrO42− and Nitrofurantoin
Source: Molecules. 2023 Jun 9;28(12):4673. doi: 10.3390/molecules28124673 (PMC10301772; doi:10.3390/molecules28124673)
Supplement: Supplementary file 1 [file molecules-28-04673-s001.zip › molecules-2412349-supplementary.pdf]

Electronic Supplementary Information (ESI) for

A Ni(II) coordination polymer as a multifunctional luminescent sensor for detection of UO_2^{2+}, $Cr_2O_7^{2-}$ and CrO_4^{2-}, and nitrofurantoin

Yun-Shan Xue, Xin-Yue Zhang, Zheng-Chen Tian, Jing-Rui Cao, Wen-Jing Wang, Ru-Xiu Tang, Jie Guo, Zheng-Hao Fei,* Jun Wang*

School of Chemistry & Environmental Engineering, Yancheng Teachers University, Yancheng 224007, China

E-mail: feizhenghao@163.com (Z.-H.F.); wjyctu@hotmail.com (J.W.)

Content

Table S1 Selected bond lengths (Å) and angles (°) for complex **1**.

Table S2 Standard deviation and detection limit calculation of complex **1** for UO_2^{2+}, $Cr_2O_7^{2-}$, CrO_4^{2-} and NFT.

Table S3 HOMO and LUMO energy levels of selected antibiotics calculated by density functional theory (DFT) at B3LYP/6-31G** level.

Scheme S1 Schematic drawing of the ligands BMIOPE and H_2MIP.

Figure S1 (a) View of 1D $[Ni(MIP)]_n$ chain in complex **1**; (b) View of 1D $[Ni(BMIOPE)]_n$ chain in complex **1.**

Figure S2 The PXRD patterns for complex **1** under different conditions.

Figure S3 The TG profile of complex **1**.

Figure S4 Solid-state excitation and emission spectra of complex **1**.

Figure S5 The HOMO and LUMO energy levels for different antibiotics.

Figure S6 Fluorescence decay curve for complex **1**.

Figure S7 Fluorescence decay curve for complex **1** in the presence of UO_2^{2+}.

Figure S8 Fluorescence decay curve for complex **1** in the presence of $Cr_2O_7^{2-}$.

Figure S9 Fluorescence decay curve for complex **1** in the presence of CrO_4^{2-}.

Figure S10 Fluorescence decay curve for complex **1** in the presence of NFT.

Table S1 Selected bond lengths (Å) and angles (°) for complex **1**.

**Complex 1**			
Ni(1)-O(1)	2.0775(15)	Ni(1)-O(2)	2.200(2)
Ni(1)-O(1)^{iv}	2.0775(15)	Ni(1)-O(2)^{iv}	2.200(2)
Ni(1)-N(1)	2.048(2)	Ni(1)-N(1)^{iv}	2.048(2)
Ni(2)-O(3)	2.0759(14)	Ni(2)-O(3)^{i}	2.0759(14)
Ni(2)-O(4)	2.1521(16)	Ni(2)-O(4)^{i}	2.1521(16)
Ni(2)-N(4)^{ii}	2.1208(19)	Ni(2)-N(4)^{iii}	2.1208(19)
O(1)-Ni(1)-O(2)	61.13(7)	O(1)-Ni(1)-O(2)^{iv}	104.24(7)
O(1)-Ni(1)-O(1)^{iv}	161.28(11)	O(2)-Ni(1)-O(1)^{iv}	104.25(7)
O(2)^{iv}-Ni(1)-O(1)^{iv}	61.13(7)	N(1)^{iv}-Ni(1)-O(1)^{iv}	92.01(7)
N(1)-Ni(1)-O(1)	92.01(7)	N(1)^{iv}-Ni(1)-O(1)	99.77(8)
N(1)-Ni(1)-O(1)^{iv}	99.77(8)	N(1)-Ni(1)-O(2)^{iv}	92.09(8)
N(1)^{iv} -Ni(1)-O(2)^{iv}	151.66(7)	N(1)^{iv} -Ni(1)-O(2)	92.08(9)
N(1)-Ni(1)-O(2)	151.66(7)	N(1)-Ni(1)-N(1)^{iv}	101.97(12)
O(3)^{i}-Ni(2)-O(4)^{i}	62.27(6)	O(3)^{i}-Ni(2)-O(4)	117.73(6)
O(3)-Ni(2)-O(4)^{i}	117.73(6)	O(3)-Ni(2)-O(4)	62.27(6)
O(3)^{i}-Ni(2)-N(4)^{ii}	92.36(7)	O(3)-Ni(2)-N(4)^{ii}	87.64(7)
O(3)-Ni(2)-N(4)^{iii}	92.36(7)	O(3)^{i}-Ni(2)-N(4)^{iii}	87.64(7)
O(4)-Ni(2)-N(4)^{iii}	88.22(7)	O(4)-Ni(2)-N(4)^{ii}	91.78(7)
O(4)^{i}-Ni(2)-N(4)^{ii}	88.22(7)	O(4)^{i}-Ni(2)-N(4)^{iii}	91.78(7)

Symmetry codes for **1**: i 1/2-x, -1/2-y, 1-z; ii 1/2-x, 3/2-y, 1-z; iii +x, -2+y, +z; iv 1-x, +y, 3/2-z.

Table S2 Standard deviation and detection limit calculation of complex **1** for UO_2^{2+}, $Cr_2O_7^{2-}$, CrO_4^{2-} and NFT.

	UO_2^{2+}	$Cr_2O_7^{2-}$	CrO_4^{2-}	NFT
1	816.440504	810.423688	796.215284	816.514443
2	816.257993	810.647530	796.455472	816.231939
3	816.197503	810.152739	796.655767	816.682595
4	816.607199	810.576772	796.315487	816.396628
5	816.362415	810.365741	796.145716	816.764256
Standard deviation (σ)	0.14391	0.17312	0.18188	0.19199
Slope (m)	1.51×10^{4}	1.27×10^{4}	1.44×10^{4}	6.18×10^{3}
Detection limit (3σ/m)	2.86×10^{-5}	4.09×10^{-5}	3.79×10^{-5}	9.32×10^{-5}

Table S3 HOMO and LUMO energy levels of selected antibiotics calculated by density functional theory (DFT) at B3LYP/6-31G** level.

	**HOMO (eV)**	**LUMO (eV)**	**Band Gap (eV)**
NFT	-6.70	-3.12	3.58
CAP	-7.24	-2.52	4.72
DTZ	-6.93	-2.32	4.61
SDZ	-6.20	-1.02	5.18
PCL	-6.49	-0.50	5.99

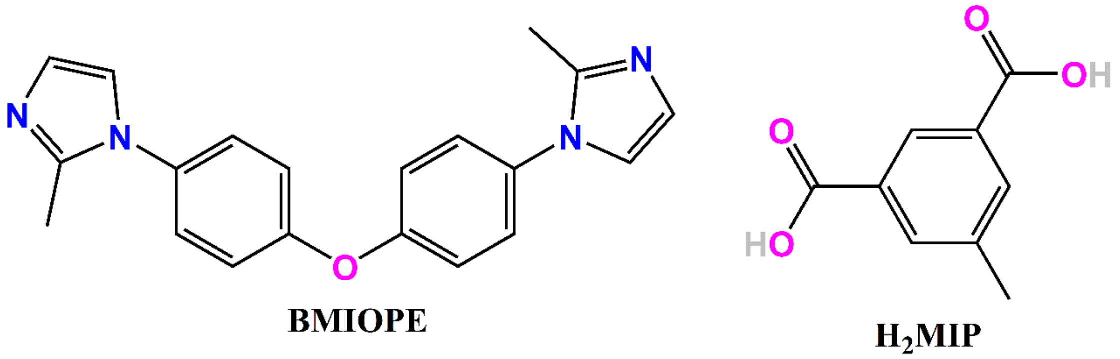

Scheme S1 Schematic drawing of the ligands BMIOPE and H2MIP.

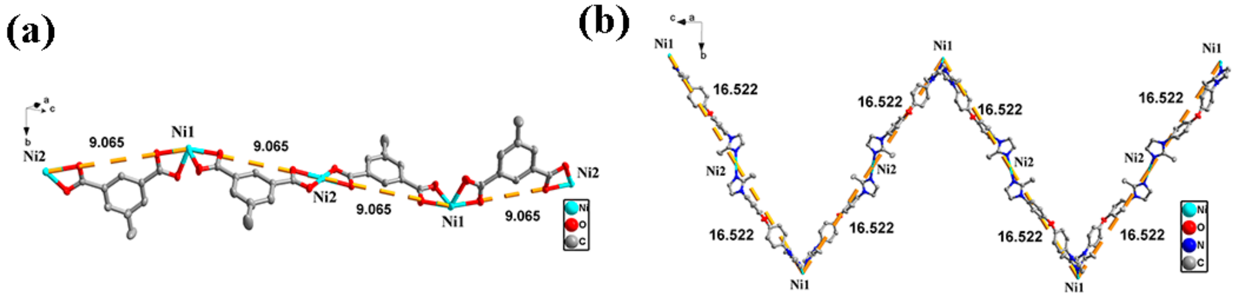

Figure S1 (a) View of 1D [Ni(MIP)]n chain in complex **1**; (b) View of 1D [Ni(BMIOPE)]n chain in complex **1**.

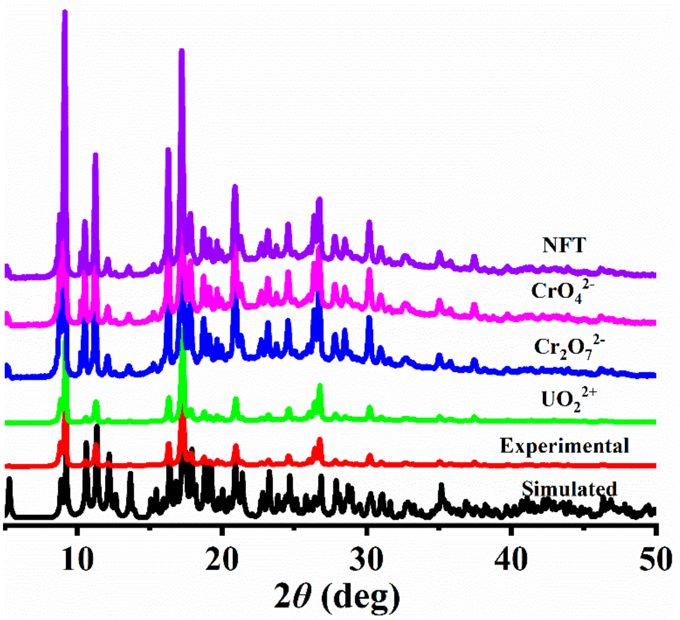

Figure S2 The PXRD patterns for complex **1** under different conditions.

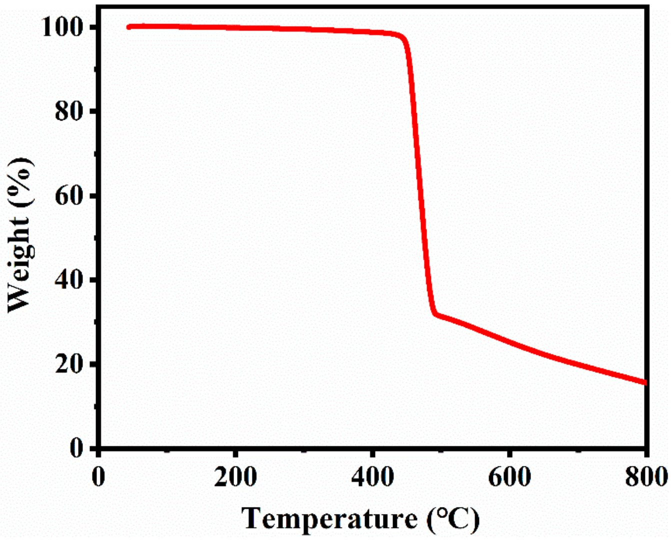

Figure S3 The TG profile of complex **1**.

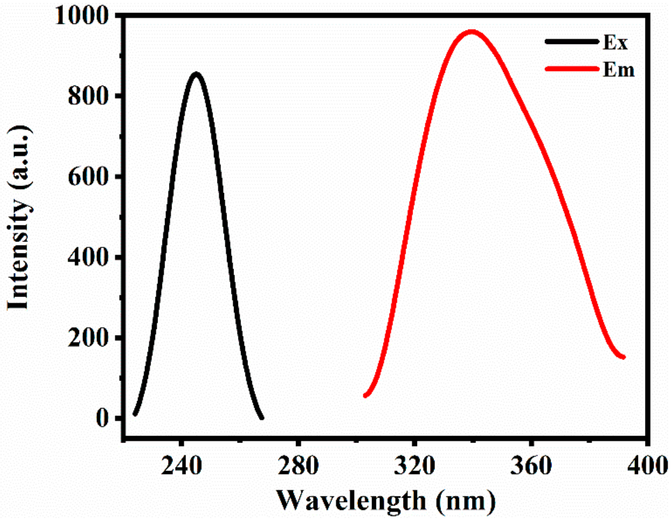

Figure S4 Solid-state excitation and emission spectra of complex **1**.

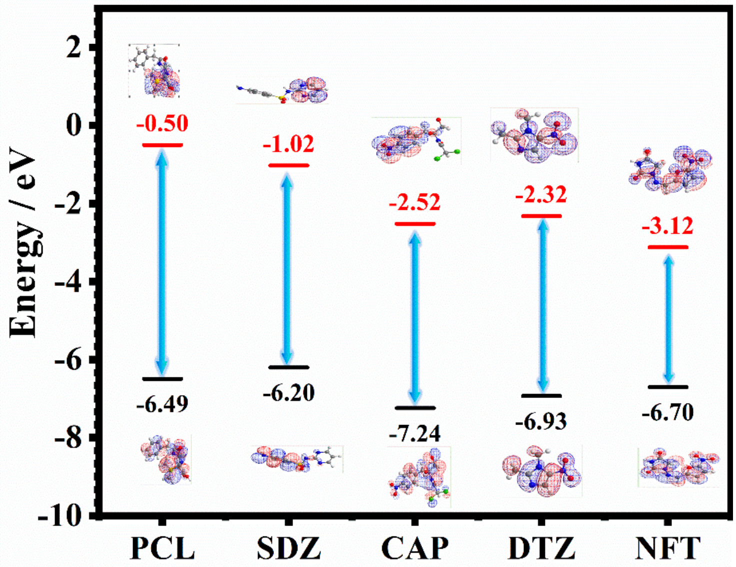

Figure S5 The HOMO and LUMO energy levels for different antibiotics.

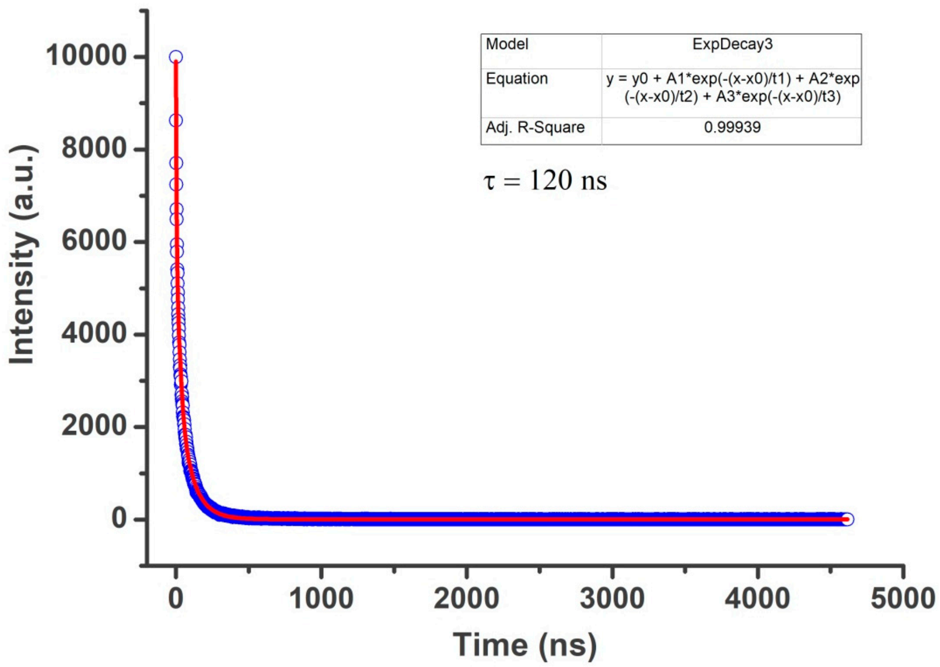

Figure S6 Fluorescence decay curve for complex **1**.

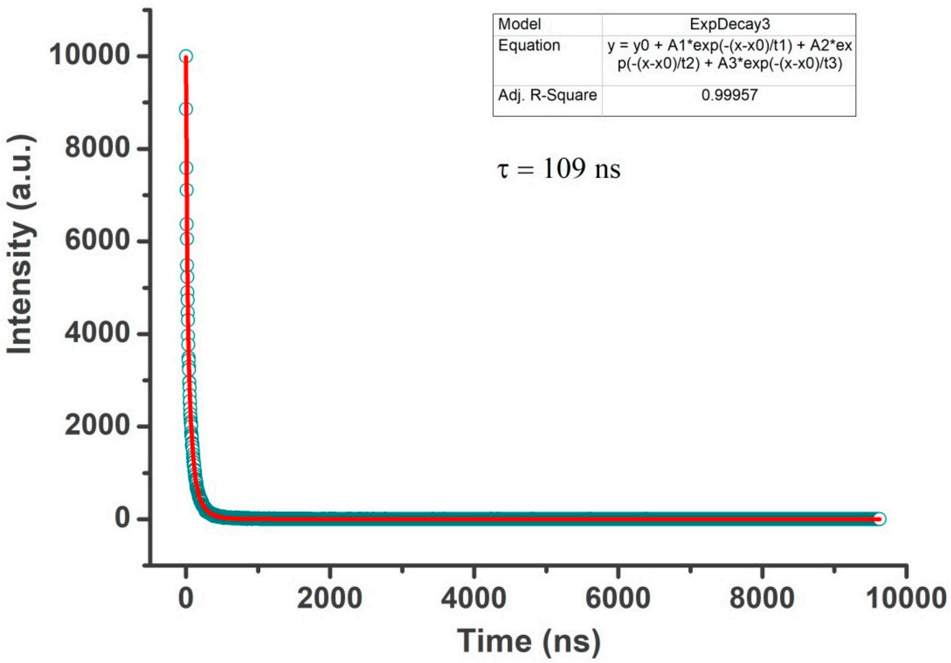

Figure S7 Fluorescence decay curve for complex **1** in the presence of $\mathbf{UO_2^{2+}}$.

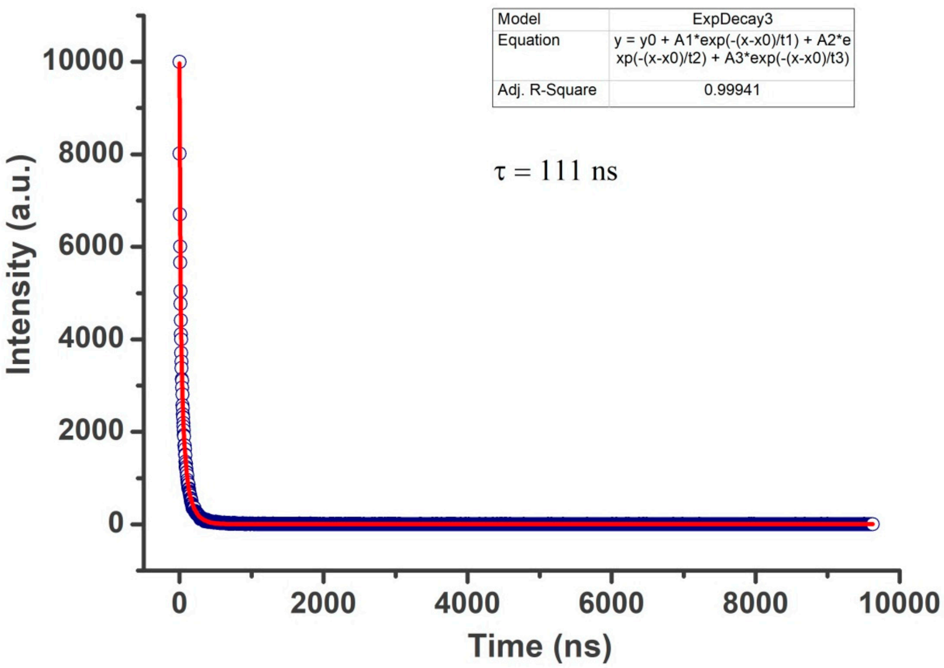

Figure S8 Fluorescence decay curve for complex **1** in the presence of $Cr_2O_7^{2-}$.

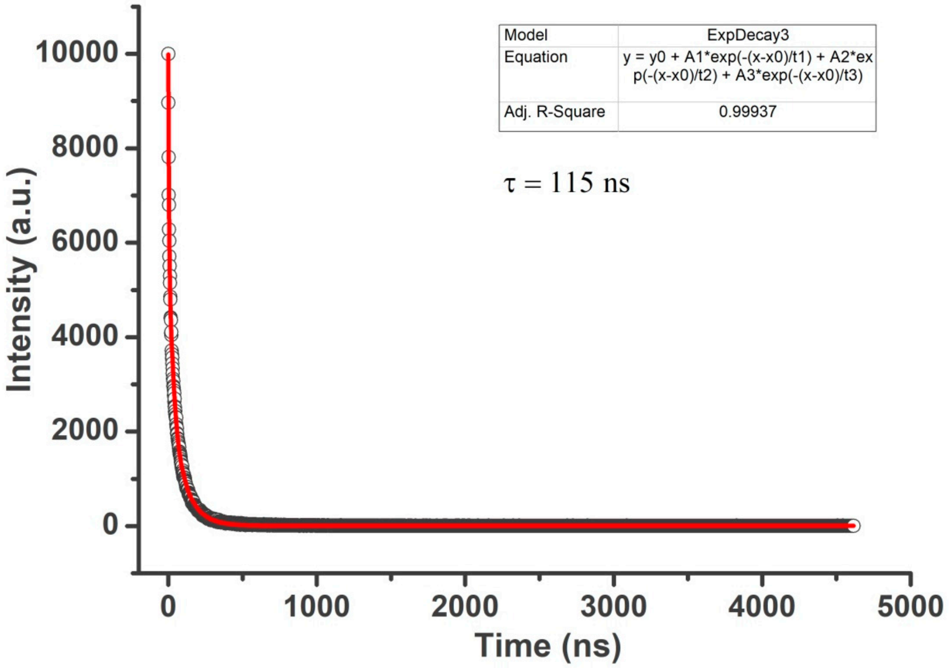

Figure S9 Fluorescence decay curve for complex **1** in the presence of CrO_4^{2-}.

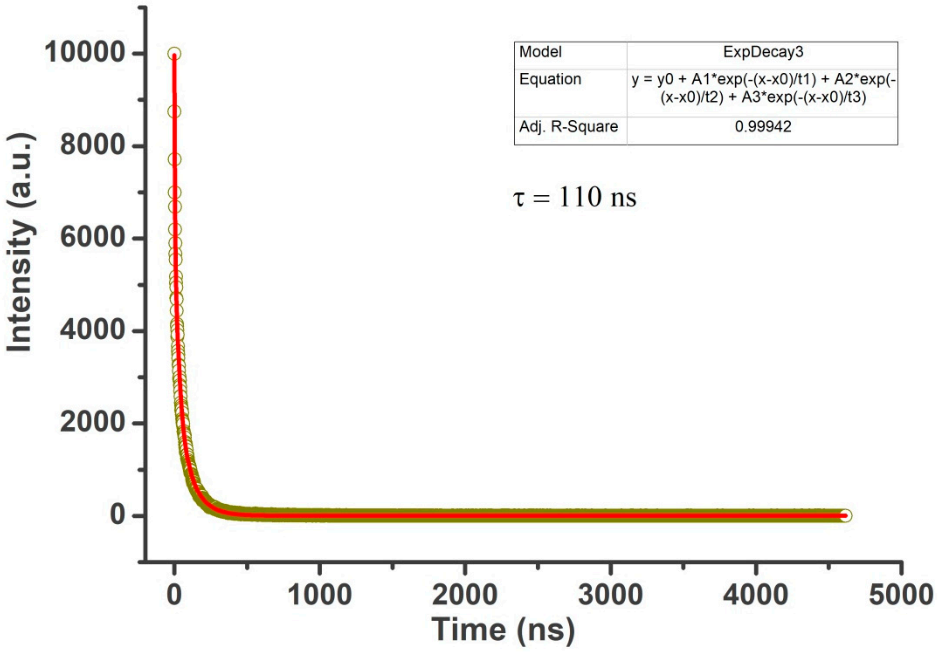

Figure S10 Fluorescence decay curve for complex **1** in the presence of NFT.
